# Thermodynamics and Crystallization Behavior of Meropenem Influenced by Solvent Composition and pH

**DOI:** 10.3390/molecules31111855

**Published:** 2026-05-28

**Authors:** Jinshen Ren, Sanli Yin, Yifu Zhang, Mingyu Chen, Na Wang, Ting Wang, Xin Huang, Hongxun Hao

**Affiliations:** 1 National Engineering Research Center of Industrial Crystallization Technology, School of Chemical Engineering and Technology, Tianjin University, Tianjin 300072, China; ren_jinshen@tju.edu.cn (J.R.); yinsanli@tju.edu.cn (S.Y.); zhangif@tju.edu.cn (Y.Z.); mychen0829@tju.edu.cn (M.C.); wang_ting@tju.edu.cn (T.W.); hongxunhao@tju.edu.cn (H.H.); 2 Engineering Research Center of Green Refining Process, Ministry of Education, Tianjin University, Tianjin 300072, China

**Keywords:** meropenem, pH-dependent solubility, molecular dynamics simulations, crystallization

## Abstract

To develop an improved crystallization process for meropenem trihydrate, an important carbapenem antibiotic, the pH-dependent solubility of meropenem trihydrate in different solvent systems was investigated using the laser monitoring dynamic method. The solubility data displayed a characteristic U-shaped profile. The solvent composition could also affect the solubility data and the isoelectric point of meropenem. The experimental solubility data were well correlated with the Tsuji model. Hirshfeld surface (HS) analysis highlighted the significance of O···H/H···O interactions in the crystal structure. Molecular electrostatic potential surfaces (MEPs) revealed complementary positive and negative regions on the meropenem molecule, enabling solute–solute aggregation through electrostatic interactions. Furthermore, molecular dynamics (MD) simulations provided deeper insights into the influence of ethanol and acetone on the solvation layer of meropenem. Additionally, the effects of pH value and solvent composition on the crystallization process of meropenem trihydrate were discussed. The crystal habit is affected by variations in solvent composition and pH. Finally, the combined effects of solvent polarity, dielectric constant, and solvation on both solubility and the crystallization process were comprehensively summarized.

## 1. Introduction

**Figure 1:**
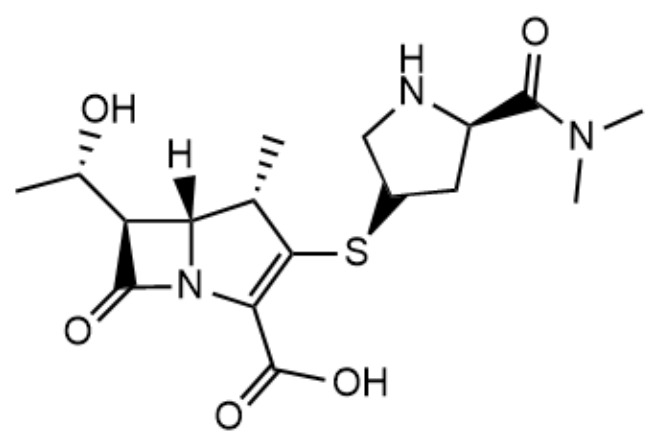
Chemical structure of meropenem.

Meropenem is a widely used carbapenem antibiotic in clinical practice, characterized by its broad-spectrum antibacterial activity and stability against β-lactamases. Its structural formula is shown in Figure 1. It exhibits notable efficacy against drug-resistant Gram-positive and Gram-negative bacteria, along with a favorable safety profile. Over the past two decades, meropenem has been regarded as a “last line of defense” against resistant bacterial infections, saving countless lives [1,2]. The trihydrate crystalline form of meropenem trihydrate (C_17_H_25_N_3_O_5_S·3H_2_O, CAS No.: 119478-56-7) is the primary pharmaceutical form currently in use. This form is produced via total chemical synthesis and requires purification through recrystallization to remove impurities and residual metal catalysts [3]. For many years, researchers have been committed to producing higher-quality meropenem trihydrate crystals at a lower cost. To develop a robust and advanced crystallization technology of meropenem, it is essential to know well its crystallization thermodynamic and kinetic properties.

Several previous studies have measured the solubility of meropenem trihydrate in water and three binary solvent systems, like water–methanol, water–acetone, and water–tetrahydrofuran, within the temperature range of 278.15 K to 303.15 K under atmospheric pressure [4,5]. However, due to the limited solubility of meropenem in water and its thermosensitivity [6]. The industrial production of meropenem typically involves a process of dissolution upon heating, followed by rapid cooling and antisolvent crystallization. However, meropenem possesses the fundamental structure of an ampholyte, and its solubility is distinctly dependent on pH, a crucial factor that has been overlooked in previous studies [4,5]. Meanwhile, industrial processes designed based on existing thermodynamic data often require substantial consumption of organic solvents to achieve satisfactory yields. Given that the solubility of ampholytes typically varies significantly with pH, exploring the relevant thermodynamic data is essential for the development of new processes [7,8]. Furthermore, gaining insights into the molecular-level mechanisms is crucial for accurately predicting solubility behavior in broader contexts. Therefore, it is both necessary and well-justified to further investigate the thermodynamic properties of meropenem. Meanwhile, a study of how solvent and pH influence the crystallization of meropenem is of paramount importance for the advancement of follow-up process design.

In this work, the laser dynamic method was employed to determine the solubility of meropenem trihydrate in water as a function of pH from 278.15 K to 298.15 K under atmospheric pressure. Meanwhile, the solubility of meropenem trihydrate in two binary solvent systems, water–ethanol and water–acetone at 278.15 K, was also assessed. Additionally, the solubility data were fitted using the Tsuji model, and the results obtained can be applied to the design and optimization of the meropenem recrystallization process. Furthermore, Hirshfeld surface analysis was conducted to examine the intermolecular interactions within the meropenem trihydrate crystal structure. Molecular dynamics (MD) simulations and molecular electrostatic potential (MEP) were utilized on molecular van der Waals (vdW) surface to analyze the interactions between meropenem molecules and solvents at the isoelectric point in binary solvent systems. The mechanism of thermodynamic solvation behavior was revealed based on experimental and simulation data. To optimize the crystallization of meropenem, the influence of variations (pH, solvents, etc.) on the quality of the crystal products was monitored in real-time using process analytical technology (PAT), based on its thermodynamic properties.

## 2. Experimental Section

### 2.1. Materials

The materials used in the experiments are listed in Table 1. Meropenem trihydrate was supplied by CSPC ZhongNuo Pharmaceutical Co., Ltd. (Shijiazhuang, China).The purity of the drug substance was determined by high-performance liquid chromatography (HPLC). The solvents employed for solubility measurements included water as the solubility-enhancing solvent, along with ethanol and acetone as antisolvents, respectively. Both the drug substance and all solvents were used as received without further purification.

### 2.2. Solubility Measurement

The solubility of meropenem trihydrate in various solvents as a function of pH was determined using the laser monitoring dynamic method [7]. In the experiment, 150 mL of solvent was first added to a 200 mL jacketed glass vessel equipped with a magnetic stirrer. The relatively large solvent volume was used to reduce interference from the crystalline water of the trihydrate crystals in the measurements. Stirring was initiated, and the temperature was controlled by a thermostatic bath circulator (Julabo CF41, JULABO Labortechnik GmbH, Seelbach, Germany) with an accuracy of ±0.02 K. The actual solution temperature was monitored using a thermometer. A laser beam was aligned to pass directly through the solution, reaching the center of the laser receiver. After temperature stabilization, the laser was activated, and the initial laser intensity was recorded as the maximum value.

All samples were weighed using an electronic analytical balance (Mettler Toledo ML204, Mettler-Toledo International Inc., Zurich, Switzerland) with an accuracy of ±0.0001 g. The pH of the solution was adjusted by adding triethylamine or hydrochloric acid and was measured using a calibrated pH meter (Mettler Toledo FiveEasy Plus, Zurich, Switzerland).

For each measurement, 0.01 g of meropenem trihydrate crystals was accurately weighed and added incrementally to the jacketed vessel. This process continued until the laser intensity stabilized at a constant value, indicating that the solute could no longer fully dissolve and that dissolution equilibrium had been reached. The temperature, pH, laser intensity, and total mass of solute added were recorded to obtain the solubility data at that specific pH.

Subsequently, a small amount of hydrochloric acid or triethylamine was added to change the pH of the solution, allowing the undissolved solute to dissolve fully and restoring the laser intensity to a value close to the initial maximum. The aforementioned steps of solute addition were then repeated to measure the solubility at the new pH condition.

To minimize degradation of meropenem during the measurement process, each solubility curve was determined through multiple measurements. The amount of degradation was determined by high-performance liquid chromatography (HPLC) to ensure that the normalized chromatographic peak area of meropenem was above 98.0%. The test was performed by an Agilent 1260 Infinity II high-performance liquid chromatograph (Agilent Technologies, Inc, Santa Clara, CA, USA) equipped with a C18 reversed-phase chromatographic column (Waters XTerra MS C18, Waters Corporation, Milford, MA, USA, 5 μm, 4.6 × 250 mm). The mobile phase consisted of a triethylamine buffer (0.1%), adjusted to pH 5.0 with phosphoric acid, and acetonitrile in a volume ratio of 93.5:6.5. The analysis was performed at 298.15 K with 220 nm UV light, and the flow rate of the mobile phase was set to 1.0 mL/min [9]. And to minimize experimental error, all data points were determined from triplicate experiments.

The molar solubility of meropenem in different solvents and pH conditions can be calculated using the following Equation (1):

(1) $$x_{A}=\frac{m_{A}/M_{A}}{m_{A}/M_{A}+\sum m_{Si}/M_{Si}}$$

where *m_A_* and *m_Si_* represent the mass of meropenem trihydrate and the solvent, respectively. *M_A_* and *M_Si_* represent the relative molecular mass of meropenem trihydrate and solvent *i*, respectively.

### 2.3. In Situ Monitoring of the Influence of Solvent Environment on Meropenem Trihydrate Crystals

In experiments involving different solvents, the mixture was first dissolved by heating and then filtered. The resulting filtrate was quenched to 278.15 K and maintained at this constant temperature. The crystallization process was monitored using an EasyViewer 100 (Mettler Toledo, Switzerland). In situ crystal images were analyzed using the Image2Chords module of the IC Vision 8.1 software (Mettler Toledo, Switzerland), enabling the measurements of turbidity, mean chord length, and the number of crystals within specified chord-length ranges. The crystal products were further imaged by scanning electron microscopy (SEM, Hitachi TM3000, Hitachi Limited, Hitachi, Japan). For comparative experiments, the same supersaturation level was controlled. In all experiments, a thermostat (Julabo CF41, JULABO Labortechnik GmbH, Seelbach, Germany) was used to maintain the temperature at 278.15 K, and the mechanical agitation rate was set at 250 rpm.

### 2.4. Powder X-Ray Diffraction

Powder X-ray diffraction (PXRD) analyses were performed on both the raw material crystal and the crystal collected after suspension under solubility test conditions. The analysis was conducted to confirm that the crystal form remained unchanged during the testing process. PXRD patterns were collected using a Rigaku D/Max2500 diffractometer (Rigaku MiniFlex600, Rigaku Coporation, Tokyo, Japan) with Cu Kα radiation (λ = 1.5405 Å). The sample was scanned continuously over a 2*θ* range from 2° to 50° at a rate of 10 °/min with a step size of 0.01°. The operating voltage and current were set at 40 kV and 100 mA, respectively.

### 2.5. Simulation Details

The crystal structure of meropenem trihydrate was obtained from the Cambridge Crystallographic Data Centre (CCDC: 1287245). Based on the meropenem molecular conformation from the single-crystal data, geometric optimization of hydrogen atoms was performed using Gaussian 09 at the M06-2X/6-311+G(d,p) level. Meanwhile, geometric optimization and frequency calculation were carried out for the solvent molecules. The MEP-mapped molecular vdW surfaces were then computed using Multiwfn 3.8 [10,11] and were visualized with VMD 1.9.3 [12]. In order to further gain deeper insights into the characteristics of the MEP surface, a visualized quantitative analysis was performed [13,14]. Additionally, Hirshfeld surface analysis and two-dimensional fingerprint plots were generated using CrystalExplorer 17.5 to examine the intermolecular interactions within the crystal structure [15].

Molecular dynamics (MD) simulations were performed using the Forcite module within Materials Studio 7.0. Both the geometry optimization and the MD simulations were conducted employing the COMPASS II force field [16]. The meropenem molecule in its zwitterionic state and the three solvent molecules were constructed separately and then subjected to geometry optimization. Subsequently, a series of periodic boxes of 40 Å × 40 Å × 40 Å were constructed using the Amorphous Cell module, in which varying numbers of meropenem molecules were placed based on the changes in meropenem solubility (Table S4), and solvent molecules were placed based on the solvent ratios [17]. These constructed systems underwent geometric optimization using the Smart method. For each solvent system, ten solvent boxes for parallel experiments were constructed, and molecular dynamics simulations were performed to ensure the reliability of the results. The MD simulations were performed using NPT ensemble at 0.1 MPa and 278.15 K for a duration of 1000 ps, with a time step of 1 fs. Temperature and pressure were controlled using a Nose thermostat and a Berendsen thermostat, respectively. The equilibrium and convergence of the system were demonstrated by the relevant parameters presented in Figure S1 [18]. Meanwhile, the electrostatic interactions were calculated using the Ewald method, while the van der Waals forces were derived using an atom-based method with a cutoff distance of 15.5 Å. A frame was recorded every 5 ps as part of the final trajectory file, resulting in a total of 200 frames generated over the 1 ns simulation time. Finally, the radial distribution function (RDF) was analyzed from the equilibrated trajectory. The hydrogen bond statistics were based on geometric criteria [19]. As the definition of a hydrogen bond is not absolute, a distance cutoff of 3.0 Å and an angle cutoff of 120° were adopted in this study [20]. For each simulation frame, a hydrogen bond was considered to be formed when the spatial positions of the relevant particles were within the defined cutoffs.

### 2.6. Thermodynamic Models

**Figure 2:**
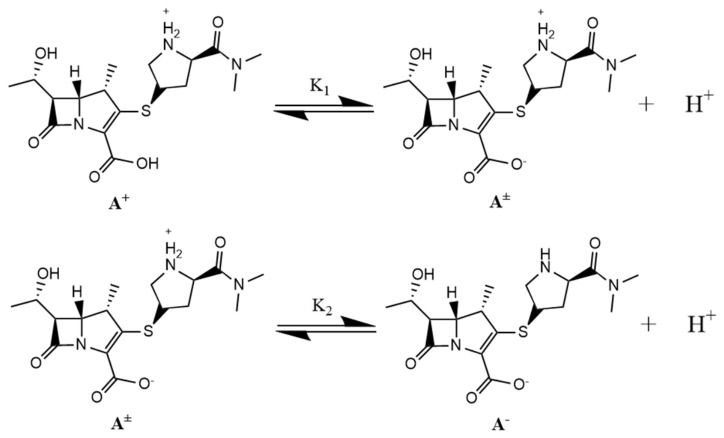
Meropenem ionization equation.

Meropenem possesses both a carboxyl group and a secondary amino group, which can be categorized as ampholytes [21]. Amino acids are a classic example of such molecules. The ionization process of meropenem is illustrated in Figure 2.

In 1977, Tsuji et al. proposed a model for calculating the influence of pH on solubility [22,23]:

(2) $$C_{T}=C_{0}(\frac{a_{H^{+}}}{K_{1}}+1+\frac{K_{2}}{a_{H^{+}}})$$

where *C_T_* represents the total solubility in mg/mL; *C*_0_ denotes the intrinsic solubility in mg/mL; *K*_1_ and *K*_2_ represent carboxyl group and amino group dissociation constants, respectively; and *a_H_^+^* indicates the hydrogen ion activity in the solution.

The total solubility of meropenem is the sum of the solubilities of the cationic, anionic, and neutral species, which can be expressed as:

(3) $$x_{A}=x_{A^{\pm }}+x_{A^{+}}+x_{A^{-}}$$

where *x_i_* represents the molar fraction solubility of meropenem and species.

The dissociation constants *K*_1_ and *K*_2_ can be calculated using the following formula:

(4) $$K_{1}=a_{A^{\pm }}a_{H^{+}}/a_{A^{+}}$$

(5) $$K_{2}=a_{A^{-}}a_{H^{+}}/a_{A^{\pm }}$$

where *a_i_* represents the activity of *i*-compound.

Based on the definition, it follows that:

(6) $$a_{H^{+}}=10^{-pH}$$

(7) $$pK_{i}=-\mathrm{lg}K_{i}$$

Based on Equation (1), the following expression can be derived:

(8) $$\begin{array}{l} \frac{x_{A}}{x_{A_{0}}}=\left(\frac{\frac{C_{T}}{M_{A}}}{\frac{C_{T}}{M_{A}}+\sum \frac{\rho_{Ti}}{M_{S}}}/\frac{\frac{C_{0}}{M_{A}}}{\frac{C_{0}}{M_{A}}+\sum \frac{\rho_{0i}}{M_{Si}}}\right)=\frac{C_{T}}{C_{0}}\left(\frac{C_{0}+\sum \frac{\rho_{0i}M_{A}}{M_{Si}}}{C_{T}+\sum \frac{\rho_{Ti}M_{A}}{M_{Si}}}\right) \end{array}$$

where $\rho_{0i}=\frac{m_{Si_{0}}}{V_{Si_{0}}}$, $\rho_{Ti}=\frac{m_{{\mathrm{Si}}_{T}}}{V_{Si_{T}}}$; *x*_A0_ represents the intrinsic molar fraction solubility; *m_Bi_*_0_ and *m_BiT_* represent the mass of the solvent at the isoelectric point pH and other pH values, respectively; *V_Bi_*_0_ and *V_BiT_* represent the volume of the solvent at the isoelectric point pH and volume at other pH values, respectively.

In dilute solutions, $C_{0}\ll \sum \frac{\rho_{0i}M_{A}}{M_{Si}},\;C_{T}\ll \sum \frac{\rho_{Ti}M_{A}}{M_{Si}}$; therefore, Equation (8) can be simplified to:

(9) $$C_{T}/C_{0}=x_{A}/x_{A_{0}}$$

By combining Equations (2), (6) and (9), the Tsuji model expressed in terms of mole fraction is obtained:

(10) $$x_{A}=x_{A_{0}}\left[10^{\left(pK_{1}-pH\right)}+10^{\left(pH-pK_{2}\right)}+1\right]$$

The isoelectric point (p*I*) of meropenem can be calculated using the following equation:

(11) $$pI=\frac{1}{2}\left({\mathrm{pK}}_{1}{+\mathrm{pK}}_{2}\right)$$

The mean deviation (*MD*) and the root-mean-square deviation (*RMSD*) are employed to assess the reliability and accuracy of the Tsuji model:

(12) $$MD=\frac{\sum_{i=1}^{N}\left|x_{i}^{\exp}-x_{i}^{cal}\right|}{N}$$

(13) $$RMSD={\left[\frac{1}{N}{\sum_{i=1}^{N}\left(x_{i}^{\exp}-x_{i}^{cal}\right)}^{2}\right]}^{1/2}$$

where *x_i_^exp^* and *x_i_^cal^* represent the experimental mole fraction and calculated mole fraction solubility, respectively. *N* stands for the number of the experimental points.

## 3. Results and Discussion

### 3.1. The Solubility of Meropenem Trihydrate in Water with Different pH

**Figure 3:**
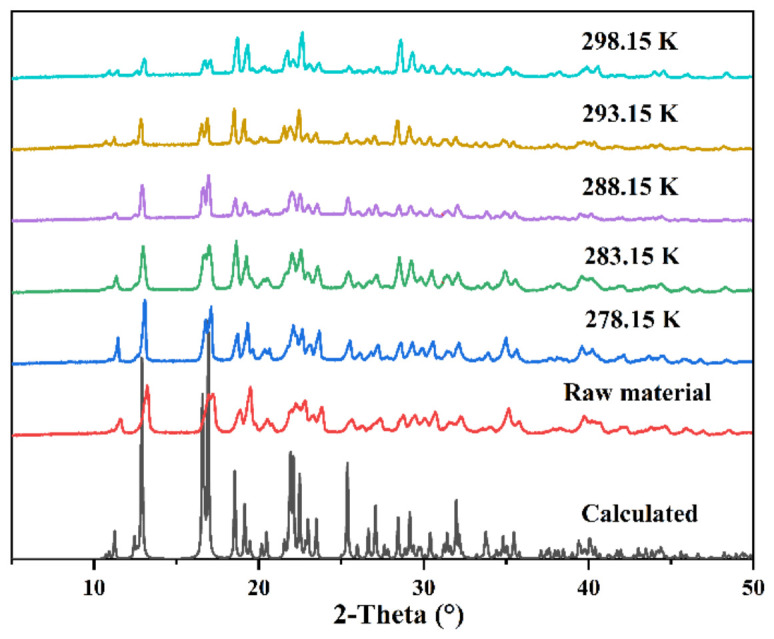
The calculated PXRD pattern was derived from the single-crystal structure (CCDC: 1287245) and the experimental PXRD patterns of the raw material and the crystals collected from water across the temperature range of 278.15 K to 298.15 K.

Before the solubility experiments, PXRD analysis was performed on the raw material crystals. And following the experiments, the remaining crystals were collected by filtration and were subjected to PXRD characterization, as shown in Figure 3. The resulting patterns are consistent with previously reported data and the simulated PXRD pattern derived from the single-crystal structure shown in Figure 3. These observations confirm that the raw material exists in the form of meropenem trihydrate crystals and indicate that no polymorphic transformation occurred during the experimental process.

**Figure 4:**
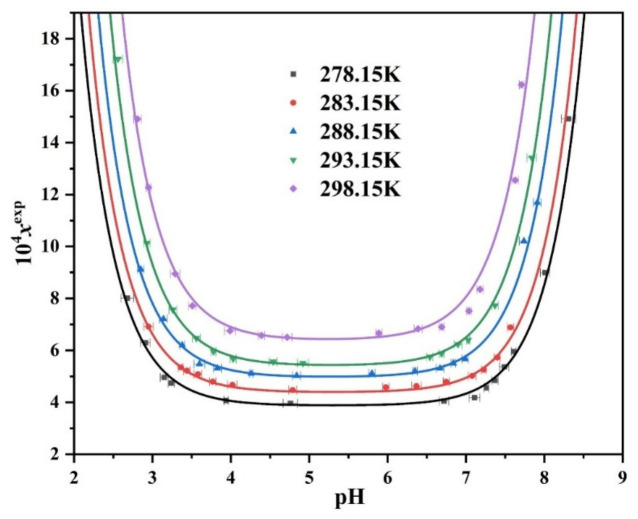
Mole fraction solubility of meropenem trihydrate in water at different pH values from 278.15 K to 298.15 K (solid lines represent the fitting curves of the Tsuji model).

The solubility of meropenem trihydrate in water at different pH values was measured under atmospheric pressure across the temperature range of 278.15 K to 298.15 K. The measured solubility data are listed in Table S1 and shown in Figure 4. In pure water, the initial meropenem solution is acidic. The results demonstrate a gradual increase in solubility with the addition of either hydrochloric acid or triethylamine, exhibiting a characteristic U-shaped curve as a function of pH, which is consistent with the fundamental behavior of ampholytes [24]. The entire solubility curve was observed to shift downward with decreasing temperature, indicating a reduction in the solubility of meropenem trihydrate at each corresponding pH value. However, as previous studies have indicated, under temperature conditions where meropenem remains relatively stable, the variation in solubility with temperature is relatively modest [4]. Notably, the solubility of meropenem trihydrate exhibits significant sensitivity to pH, with a considerable increase in solubility occurring within a narrow pH range. This pronounced enhancement in solubility may allow for a substantial reduction in solvent consumption.

The experimental solubility data were fitted using Tsuji model. The calculated data are shown in Table S1. The parameters of the Tsuji model and the p*I* values at different temperatures are presented in Table 2. From Table S1 and Table 2, it can be seen that the calculated data demonstrate a satisfactory correlation with the experimental results.

### 3.2. The Solubility of Meropenem Trihydrate in Water–Ethanol/Acetone

**Figure 5:**
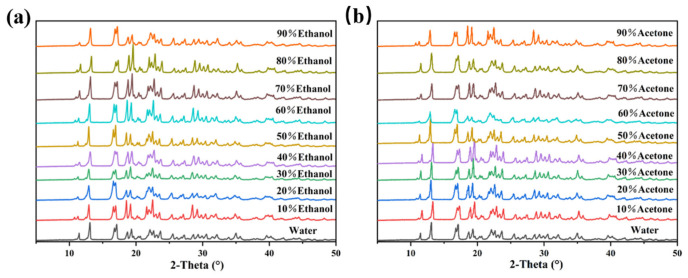
PXRD patterns of the crystals collected after solubility measurements in binary solvent systems with different volume fractions ratios of anti-solvents: (**a**) water–ethanol and (**b**) water–acetone.

Furthermore, the solubility of meropenem trihydrate as a function of pH was measured in water + ethanol/acetone mixed solvent systems with varying volume ratios under atmospheric pressure at 278.15 K. The residual crystals obtained after testing were collected by filtration and were characterized using PXRD, with the data shown in Figure 5. The resulting patterns showed no shift in peak positions, thus confirming the absence of polymorphic transformation.

**Figure 6:**
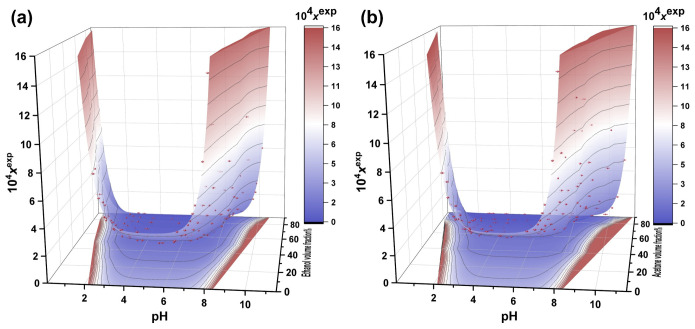
Mole fraction solubility of meropenem trihydrate in binary solvent system (with ethanol or acetone volume fractions ranging from 10% to 90%) as a function of pH: (**a**) water–ethanol, (**b**) water–acetone (three-dimensional surface fitting using the Tsuji model).

The mole fraction solubility of meropenem trihydrate as a function of pH in two binary solvent systems at 278.15 K is listed in Tables S2 and S3, and shown in Figure 6a and Figure 6b, respectively. It can be observed that the solubility decreases with the addition of ethanol or acetone.

During the measurement process, it was observed that, with the gradual addition of ethanol or acetone, the p*K*_1_ value shifted to lower values. This resulted in a continuous decrease in the minimum achievable pH when a sufficient number of data points were tested. Previous reports have indicated that meropenem exhibits lower stability in solutions with lower pH [9,25]. During the experiments, it was observed that the degradation rate of the solute accelerated considerably in solutions with extremely low pH. This hindered the acquisition of accurate thermodynamic data, and such degradation is also undesirable in practical operations. Therefore, throughout the measurements, the pH was consistently maintained at a value not lower than 2.5 to keep the degradation rate within a controllable range.

The experimental solubility data in mixed solvents were also fitted using Tsuji model. The calculated data are shown in Tables S2 and S3. The model parameters, p*I*, *RMSD*, and *MD* values obtained from fitting the Tsuji model to the two binary solvent systems are summarized in Table 3 and Table 4, respectively. Both systems show satisfactory correlation with the experimental data, as evidenced by the RMSD value being below 3.37 × 10^5^ and *MD* value below 2.21 × 10^5^ for water–ethanol system and the *RMSD* value below 3.74 × 10^5^ and *MD* value below 2.23 × 10^5^ for water–ethanol system, respectively. Compared with ethanol, the p*K*_1_ and p*K*_2_ parameters for acetone exhibit more significant variations, from 2.618 to 1.958 for p*K*_1_ and 7.962 to 8.272 for p*K*_2_, respectively. This can be attributed to the lower polarity of acetone, that makes it more difficult for the neutral electrolyte to gain or lose proton as pH changes [26].

### 3.3. Hirshfeld Surface Analysis

**Figure 7:**
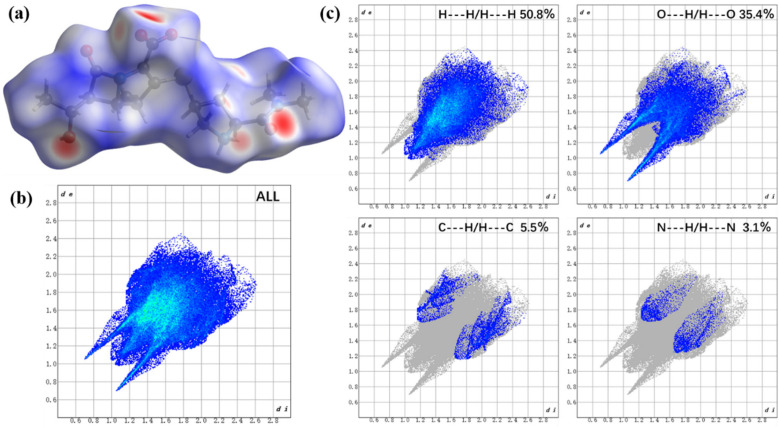
(**a**) Hirshfeld surface map and (**b**,**c**) fingerprint plots for meropenem trihydrate.

Hirshfeld surface analysis provides effective visualization of weak intermolecular interactions. Using CrystalExplorer 17.5, the Hirshfeld surfaces and corresponding 2D fingerprint plots were generated for the meropenem molecule within the single crystal structure of meropenem trihydrate (CCDC: 1287245), and the results are shown in Figure 7. The analysis reveals that there are strong interactions between the -COO^−^, -OH, =O, and -NH_2_^+^ groups and surrounding molecules. The 2D fingerprint plots indicate that H···H contacts are the most abundant, constituting 50.8% of the surface, followed by O···H/H···O and C···H/H···C interactions, accounting for 35.4% and 5.5% of the contacts, respectively. These findings collectively indicate that electrostatic interactions resulting from proton transfer and hydrogen bonds involving electronegative nitrogen and oxygen atoms play a dominant role in molecular packing within the crystal [27]. The dissolution of the crystal requires solvent molecules to disrupt these interactions, while crystal formation relies on these specific contact sites to facilitate ordered molecular rearrangement. The two distinct sharp spikes observed in the 2D fingerprint plots correspond to O···H/H···O contacts, among which interactions involving -COO^−^, -OH and -NH_2_^+^ groups constitute a significant portion. This underscores the crucial role of electrostatic forces between oppositely charged groups in stabilizing the crystal structure [28].

### 3.4. Solution Speciation of Meropenem

By calculating p*K*_1_ and p*K*_2_ at different pH levels, the activity of various meropenem species (*a_A_^±^*, *a_A_^+^*, *a_A_^−^*) in solution can be determined.

Assuming that the sum of the activity coefficients for the three distinct meropenem species equals unity, as shown in Equation (14):

(14) $$a_{A^{\pm }}+a_{A^{+}}+a_{A^{-}}=1$$

Based on Equations (3)–(5) and (14), the following expression can be derived:

(15) $$a_{A^{\pm }}=\frac{K_{1}a_{H^{+}}}{K_{1}K_{2}+K_{1}a_{H^{+}}+{a_{H^{+}}}^{2}}$$

(16) $$a_{A^{+}}=\frac{a_{H^{+}}a_{A^{\pm }}}{K_{1}}$$

(17) $$a_{A^{-}}=1-a_{A^{\pm }}-a_{A^{+}}$$

**Figure 8:**
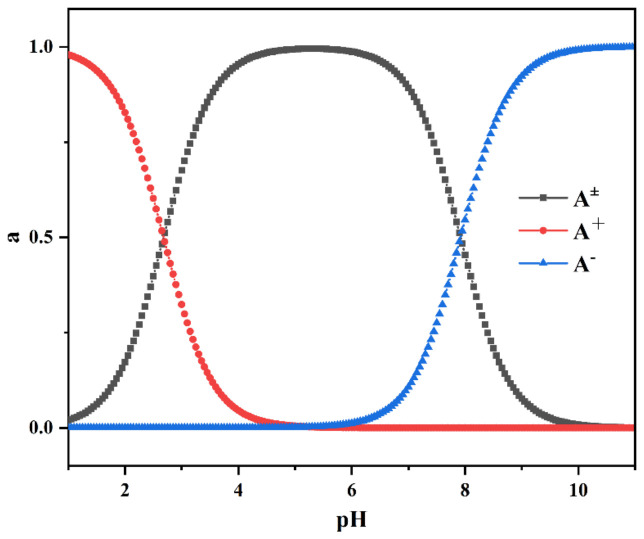
Activity distribution profiles of the three meropenem species in water as a function of pH.

Therefore, the activity distribution of different meropenem species at various pH values can be calculated using the equations. Taking the water at 278.15 K as an example, the distribution profiles are plotted in Figure 8.

From Figure 8, it can be seen that meropenem, in its cationic or anionic state, carries charges that generate considerable electrostatic repulsion between solute molecules, thereby inhibiting solute aggregation and leading to higher solubility [29,30]. Conversely, at the isoelectric point, in its zwitterionic state, where the net charge of the meropenem molecule is zero, the solubility decreases [31].

### 3.5. Molecular Electrostatic Potential Surface (MEPs)

At the isoelectric point, the meropenem molecule carries a net charge of zero while retaining localized charges. At this corresponding pH, solubility is minimized due to the weakened repulsive electrostatic interactions. This condition is commonly utilized as the endpoint for pH adjustment in crystallization processes [32,33]. Therefore, investigating and predicting the molecular mechanisms at the isoelectric point is of particular importance.

**Figure 9:**
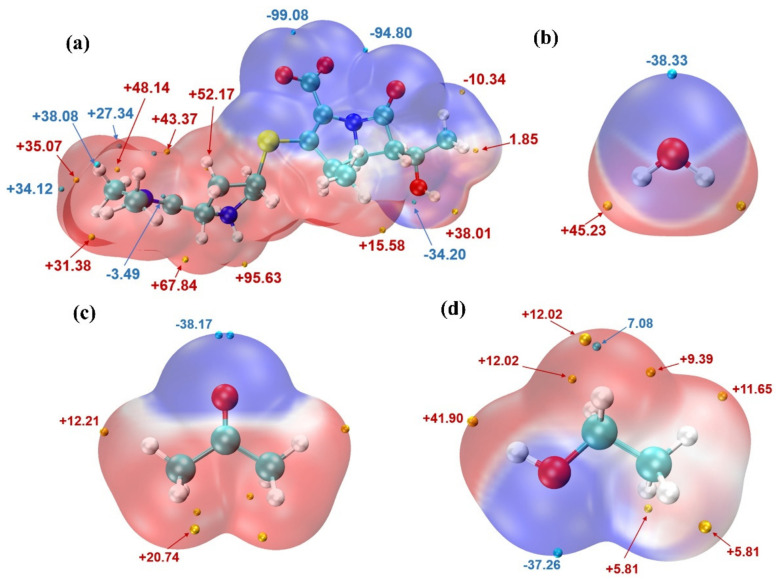
Molecular electrostatic potential surface of (**a**) meropenem, (**b**) water, (**c**) acetone, (**d**) ethanol (Blue and red are employed to represent regions of negative and positive electrostatic potential, respectively, with deeper color intensity indicating greater absolute values. Yellow points denote local maxima, while cyan points mark local minima).

The surface electrostatic potential distribution of meropenem at its isoelectric point was analyzed using MEP surface. The obtained MEP surface is shown in Figure 9a. The results demonstrate that the molecule exhibits an electrostatic potential minimum of −99.08 kcal/mol near the -COO^−^ group, representing the most electronegative region, while the molecule displays a maximum value of +95.63 kcal/mol around the -NH_2_^+^ group, indicating the most electropositive region. These extreme values typically represent the most probable sites for intermolecular interactions. The overall molecular surface reveals distinctly segregated regions of positive and negative electrostatic potentials, suggesting that meropenem molecules could potentially associate through electrostatic interactions. Additionally, MEP surface for water, ethanol, and acetone were also generated to further analyze the mechanisms underlying solute–solvent interactions. Figure 9b, c and d display the MEP surfaces of the three solvent molecules, respectively. For water, an electrostatic potential minimum of −38.33 kcal/mol is located near the oxygen atom, while a maximum of +45.23 kcal/mol is found near the hydrogen atoms. Both ethanol and acetone exhibit their electrostatic potential minima near the oxygen atoms, with values of −37.26 kcal/mol and −38.17 kcal/mol, respectively.

**Figure 10:**
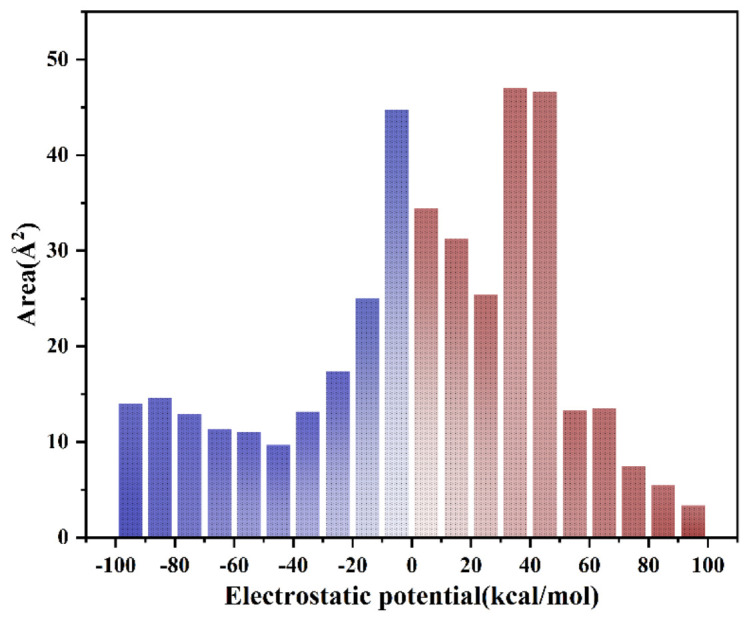
Quantitative analysis for the molecular electrostatic potential surface of meropenem.

The surface area corresponding to different MEP intervals was statistically analyzed at 10 kcal/mol increments using Multiwfn 3.8 (Figure 10). Generally, more complementary intermolecular electrostatic potentials lead to stronger generated electrostatic interactions [34,35]. The area distribution of the MEP surfaces of meropenem exhibits partial similarity, while a distinct protrusion is observed in the 30–50 kcal/mol region. Consequently, by considering both the numerical values and the distribution area of the solute’s surface electrostatic potential, a preliminary understanding of microscopic solute–solvent interactions can be established [36].

### 3.6. Molecular Dynamics Simulation

MD simulations of meropenem at its isoelectric point were performed at different solvent ratios to obtain more detailed molecular-level insights. The radial distribution function analysis was employed to characterize the probability distribution of selected particles around reference atoms during the simulations. Typically, the peak positions in RDF curves can be utilized to analyze interaction types between particle pairs and to examine solvation shell distributions [37,38].

**Figure 11:**
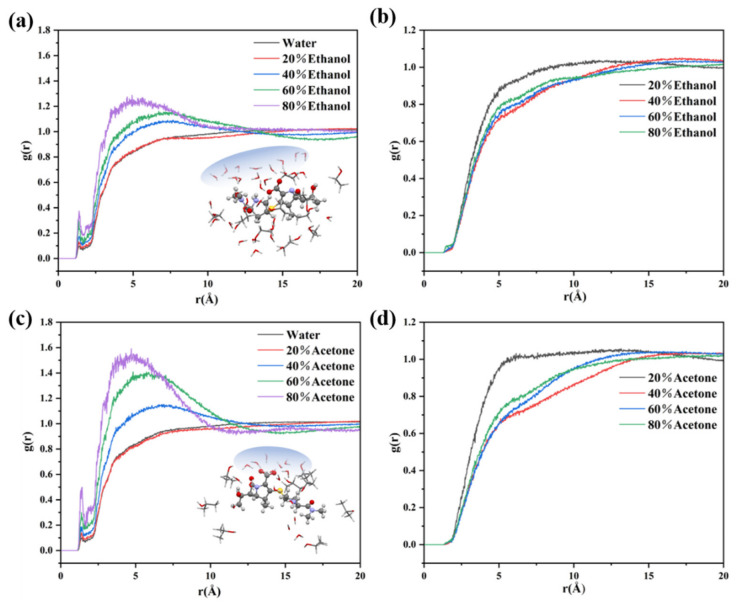
RDF analysis of binary solvent: (**a**,**b**) water–ethanol systems RDF curves of meropenem with water and ethanol, and (**c**,**d**) water–acetone systems RDF curves of meropenem with water and acetone. (The insets in (**a**,**c**) show the clusters formed by solute molecules at the center of the simulation box in the final snapshot of the MD simulation with an antisolvent volume fraction of 60%. The blue circles are used to emphasize the distribution areas of water molecules).

The RDF results for meropenem in the presence of solvent molecules at different solvent ratios are shown in Figure 11. Figure 11a,c consistently show two characteristic peaks for water molecules at approximately 1.4 Å and 5.0 Å. This demonstrates the existence of well-defined solvation shells around the solute formed through weak interactions. In contrast, Figure 11b,d reveal relatively weak interactions between the antisolvent and solute molecules, as evidenced by the absence of distinct first solvation shells and the lower molecular density in the second solvation shell compared to the bulk phase. These RDF profiles confirm the non-uniform distribution of different solvent components around meropenem molecules, with distribution patterns consistent with predictions based on MEP surfaces and electrostatic complementarity principles. Notably, the water peak at around 5.0 Å progressively intensifies with an increasing antisolvent proportion, while the molecular density shows a greater deviation from the bulk phase. This observation suggests that antisolvent molecules may promote reorganization of water molecules at the meropenem surface, potentially facilitating formation of new hydrogen-bonding networks [39,40].

The most positive and most negative regions of the MEP were further investigated, which are attributed to proton transfer between groups resulting in charged functional groups. Based on the obtained RDF profiles shown in Figure S2, it can be observed that strong ion–dipole interactions form between the charged groups and water molecules. The formation of solute–solvent clusters, alongside the addition of antisolvent, demonstrates an enhanced ability to sequester water molecules from the bulk solvent. This variation highlights the pronounced influence of charged groups on the solvation process of meropenem [41]. Further statistical analysis of hydrogen bonds during the molecular dynamics simulation was conducted, and the results are presented in Figure S3. It can be observed that with increasing antisolvent ratio, the number of hydrogen bonds formed between meropenem molecules and water decreases, while that formed between meropenem and the antisolvent increases. However, the number of hydrogen bonds formed between the charged group -COO^−^ and water remained almost unchanged when the antisolvent ratio was below 80%. This further demonstrates the heterogeneity of the solvation layer. Moreover, it was further observed that upon reaching the 80% ratio, although the number of hydrogen bonds between -COO^−^ and water initially decreased markedly, it gradually increased as the simulation proceeded. This corroborates that the change in solvent microstructure enables the charged groups to capture solvent molecules more readily.

In summary, the solvation structure of meropenem can be described as follows: with the addition of antisolvent, water and antisolvent molecules form a new solvent structure, thereby affecting solute–solvent interactions. Due to strong specific interactions, water molecules form partially denser hydration layers at particular regions on the meropenem molecular surface.

### 3.7. Effect of Solvent Composition on the Crystallization Process

**Figure 12:**
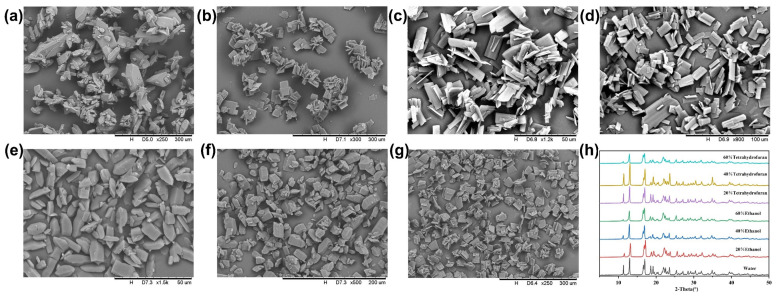
SEM images of crystal products obtained from crystallization in solvents with different volume fractions: (**a**) pure water, (**b**) 20% ethanol, (**c**) 40% ethanol, (**d**) 60% ethanol, (**e**) 20% tetrahydrofuran, (**f**) 40% tetrahydrofuran, (**g**) 60% tetrahydrofuran, and (**h**) PXRD pattern of the crystalline product.

Crystallization is a complex process influenced by factors such as conformational changes in flexible molecules and solute–solvent interactions [42]. Inspired by the aforementioned molecular simulations, it is hypothesized that alterations in solvent composition may affect solute clusters, subsequently influencing the crystallization pathway. To analyze the resulting variations in crystal products, PAT was employed in the crystallization experiments [43]. In this section, the supersaturation before nucleation was set to 5 for all crystallization experiments. Thermodynamic data are based on the research of this article and previously reported data [4]. The results are shown in Figure 12 and Figures S4–S10.

For the crystallization experiments in pure water, crystal images and in-line monitoring data are presented in Figure 12a and Figure S4, respectively. The induction period was approximately 30 min, followed by nucleation that persisted until around the fifth hour. During the equilibration stage, approximately 4800 nuclei were captured in the images. The final crystal products exhibited a broad particle size distribution, which can be attributed to the significant variation in nucleation times. In the solutions containing ethanol, crystal images and in-line monitoring data are shown in Figure 12b–d and Figures S5–S7, respectively. The addition of ethanol altered the crystal habit, resulting in reduced crystal thickness and a morphology transformation from tabular to plate-like. This change is unfavorable for the downstream processing of the final product and could increases the potential for aggregation. In the three solvent systems with varying ethanol ratios, approximately 6000 nuclei were identified from images captured at 1 h, 3 h, and 2 h, respectively. Furthermore, the curve rises more steeply than that observed in pure water. This demonstrates that ethanol also accelerated the rate of nucleation, leading to a more uniform crystal size distribution, which is advantageous for producing smaller, more controlled crystals.

The water–tetrahydrofuran solvent system is commonly used in the crystallization process of meropenem and exhibits a thermodynamic trend similar to that of the water–acetone system, namely a decrease in the solubility of meropenem trihydrate with an increasing proportion of organic solvent [4]. Both tetrahydrofuran and acetone possess one oxygen atom that can act as a hydrogen bond donor, combining with water molecules to form aggregates, thereby disrupting the hydrogen bond network among water molecules [44,45]. This is similar to the change in the water–acetone solvent network discussed in the molecular simulations. To further investigate the influence of this phenomenon on the nucleation process of meropenem, the crystallization process of meropenem in the water–tetrahydrofuran system was analyzed using PAT. Crystal images and in-line monitoring data for solutions containing tetrahydrofuran are presented in Figure 12e–g and Figures S8–S10, respectively. When the tetrahydrofuran volume fraction was below 40%, the crystal habit remained tabular. Similar to ethanol, tetrahydrofuran can also accelerate the process of nucleation. At approximately 40 min, 3 h, and 30 min, respectively, around 6000 crystals were captured in the images. Under same testing time, the nucleus counts observed was higher than that recorder in pure water. Notably, at 20% tetrahydrofuran, a large number of nuclei formed within tens of minutes. However, at 60% tetrahydrofuran, the habit changed to plate-like. Figure 12h presents the PXRD pattern of the obtained crystal product. The observations confirm that adjusting the solvent ratio did not induce polymorphic transformation.

The variable morphology and particle size of meropenem crystals create a customizable foundation for the powder form used in clinical applications. Strict control over the average particle size distribution is critical to ensure rapid dissolution. Therefore, by adjusting the solvent composition, the crystal habit of meropenem can be modified. Simultaneously, controlling the number of nuclei during crystallization allows for precise tuning of the final product’s particle size distribution. This dual-control approach provides greater flexibility in practical process design.

### 3.8. Effect of pH on the Crystallization Process

**Figure 13:**
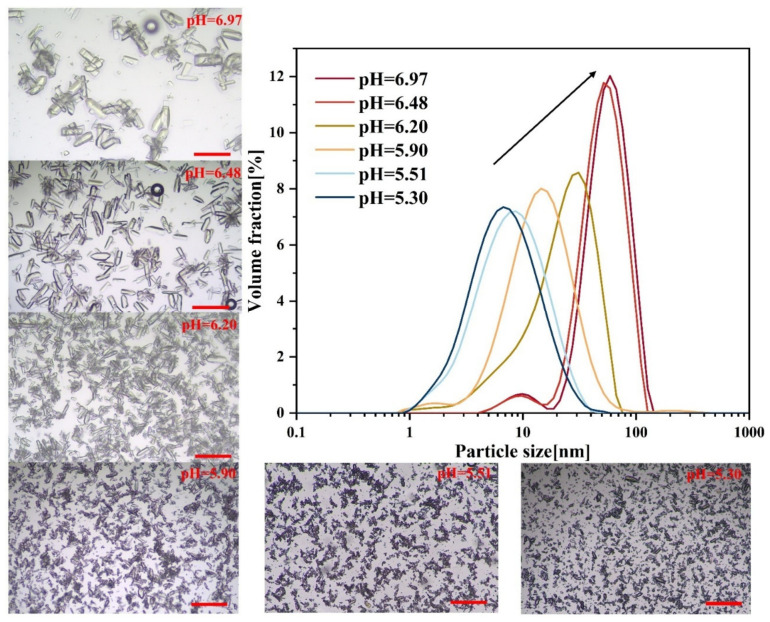
Optical micrographs and particle size distribution of crystals crystallized at different pH values (The red scale bar represents 100 μm).

In isoelectric point crystallization, pH is a key process parameter that markedly affects the quality of crystalline products. Rational control of pH is crucial for producing high-quality crystals [46]. In this section, the initial mole fraction concentration for all crystallization experiments was 4.101 × 10^−3^. Within the pH range investigated in the experiments, the solubility increased with increasing pH. Based on the thermodynamic data, the supersaturation values corresponding to the studied pH values from high to low are 9.52, 10.22, 10.39, 10.48, 10.53, and 10.54, respectively. In the particle size distribution diagrams and optical microscopy images presented in Figure 13, considerable differences in crystal particle size distribution can be observed with changes in pH. Compared with the slight variation in supersaturation, the change in particle size distribution is more marked, particularly when the pH is below 6.48. This indicates that, under the same initial concentration, the nucleation rate can be affected by adjusting pH, and the particle size distribution of the final product can be further regulated accordingly. Notably, it was observed that a greater deviation from the isoelectric point corresponds to a larger crystal size, likely due to the effect of changes in solute charge.

### 3.9. Mechanism of Dissolution and Crystallization of Meropenem Trihydrate

**Figure 14:**
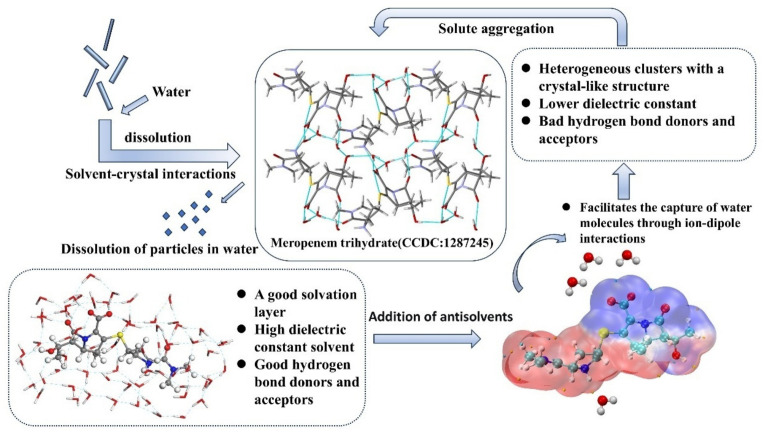
Schematic diagram of the dissolution mechanism for meropenem trihydrate (Clusters of meropenem in water were extracted from the final snapshot of the MD simulation).

Based on the aforementioned analyses, a dissolution and crystallization mechanism for meropenem trihydrate at its isoelectric point is proposed, as illustrated in Figure 14. In pure water, the high dielectric constant and strongly polar protic nature of the solvent provide effective electrostatic screening and promote solvation. However, with the addition of antisolvents, the overall dielectric constant of the solution decreases, facilitating the aggregation of solute molecules through enhanced electrostatic interactions. Concurrently, the introduction of organic solvents disrupts the uniform hydration layer, causing water molecules to preferentially localize near the -COO^−^ and -NH_2_^+^ groups via ion–dipole interactions, thereby forming an inhomogeneous solvation layer. Such cluster formation aligns with the intermolecular interactions emphasized by Hirshfeld surface analysis and corresponds to the fundamental structural units of meropenem trihydrate crystals. This structural correspondence accelerates the desolvation process, thereby favoring the aggregation of meropenem molecules in solution over their dissolution and dispersion in the solvent [47].

## 4. Conclusions

The solubility of meropenem trihydrate as a function of pH was determined in water from 278.15 K to 298.15 K and in two binary solvent systems (water–ethanol and water–acetone) at 278.15 K using the laser monitoring dynamic method. The experimental data were fitted using the Tsuji model, which yielded satisfactory correlation results. The model parameters p*K*_1_ and p*K*_2_ exhibited systematic variations in response to changes in temperature or solvent polarity. The dissolution mechanism was further elucidated through molecular simulations. Hirshfeld surface analysis revealed that crystal formation and stability are influenced by interactions among -COO^−^, -OH, and -NH_2_^+^ groups as well as water molecules. MEP surfaces demonstrated that solute–solute aggregation can be driven by electrostatic interactions. MD simulations indicated that ethanol and acetone promote localized association of water molecules near -COO^−^ and -NH_2_^+^ groups. Moreover, the effects of solvent composition and pH on the meropenem crystallization process were investigated. It was found that the solvent composition exerts a significant influence on the nucleation rate and the crystal habit, primarily due to alterations in the dielectric constant of the solvent. This work is expected to provide valuable thermodynamic data and control factors for the design and development of meropenem crystallization processes.

## Figures and Tables

**Table 1:** Sources and mass purity of materials used in the experiment.

Chemical Name	CAS No.	Source	Mass Purity
Meropenem trihydrate	119478-56-7	CSPC ZhongNuo Pharmaceutical Co., Ltd.	≥99.5%
Water	7732-18-5	Tianjin Yuanli Chemical Co., Ltd. (Tianjin, China)	for HPLC
Acetone	67-64-1	Tianjin Jiangtian Chemical Co., Ltd. (Tianjin, China)	≥99.7%
Ethanol	64-17-5	Tianjin Kmart Chemical Technology Co., Ltd. (Tianjin, China)	≥99.7%
Tetrahydrofuran	109-99-9	Tianjin Kmart Chemical Technology Co., Ltd.	≥99.5%
Triethylamine	121-44-8	Tianjin Komio Chemical Reagent Co., Ltd. (Tianjin, China)	≥99.7%
Hydrochloric acid	7647-01-0	Tianjin Yuanli Chemical Co., Ltd.	for HPLC
Acetonitrile	75-05-8	Tianjin Pursunret Biomedical Technology Co., Ltd. (Tianjin, China)	≥99.9%
Phosphoric acid	7664-38-2	Tianjin Kmart Chemical Technology Co., Ltd.	for HPLC

**Table 2:** Model parameters, p*I*, *RMSD*, and *MD* for Tsuji model used for fitting experimental solubility data in water across 278.15–298.15 K.

Temperature/K	10^4^*x_A_*_0_	p*K*_1_	p*K*_2_	p*I*	10^5^*RMSD*	10^5^*MD*
278.15	3.872	2.681	7.918	5.30	4.40	2.57
283.15	4.382	2.712	7.893	5.30	1.33	0.896
288.15	4.961	2.763	7.778	5.27	2.08	1.17
293.15	5.402	2.863	7.689	5.28	2.95	1.68
298.15	6.376	2.912	7.584	5.25	5.17	3.54

**Table 3:** Model parameters, p*I*, *RMSD*, and *MD* for Tsuji model used for fitting experimental solubility data in water–ethanol at 278.15 K.

	10^4^*x_A_*_0_	p*K*_1_	p*K*_2_	p*I*	10^5^*RMSD*	10^5^*MD*
10%	2.998	2.664	7.928	5.30	3.37	2.21
20%	2.229	2.614	7.942	5.28	1.54	1.26
30%	1.474	2.542	7.957	5.25	2.81	1.38
40%	1.032	2.496	7.993	5.24	1.51	1.12
50%	0.5989	2.418	8.001	5.21	0.982	0.773
60%	0.5072	2.378	8.042	5.21	0.663	0.454
70%	0.4062	2.331	8.072	5.20	1.00	0.790
80%	0.2464	2.283	8.126	5.20	1.78	0.917
90%	0.05906	2.182	8.168	5.18	1.20	0.84

**Table 4:** Model parameters, p*I*, *RMSD*, and *MD* for Tsuji model used for fitting experimental solubility data in water–acetone at 278.15 K.

Acetone Volume Fraction	10^4^*x_A_*_0_	p*K*_1_	p*K*_2_	p*I*	10^5^*RMSD*	10^5^*MD*
10%	3.069	2.618	7.962	5.29	1.74	1.34
20%	2.483	2.542	7.978	5.26	1.27	0.903
30%	1.791	2.492	8.029	5.26	1.96	1.28
40%	1.032	2.409	8.061	5.24	3.74	2.23
50%	0.9124	2.322	8.081	5.20	1.79	1.38
60%	0.6434	2.221	8.138	5.18	2.17	1.39
70%	0.3782	2.163	8.168	5.17	0.587	0.405
80%	0.08424	2.070	8.207	5.14	0.884	0.564
90%	0.01279	1.958	8.272	5.12	1.23	0.736

## Data Availability

The original contributions presented in this study are included in the article/Supplementary Material. Further inquiries can be directed to the corresponding author(s).

## Supplementary Materials

The following supporting information can be downloaded at: https://www.mdpi.com/article/10.3390/molecules31111855/s1, Figure S1. The total energy, temperature and potential energy change over the simulation time. Table S1. The mole fraction solubility of meropenem trihydrate in water from 278.15 K to 298.15 K determined by experiments and calculated by Tsuji model. Table S2. Experimental and Tsuji model calculated mole fraction solubility of meropenem trihydrate in water-ethanol at 278.15 K. Table S3. Experimental and Tsuji model calculated mole fraction solubility of meropenem trihydrate in water-acetone at 278.15 K. Table S4. The number of meropenem molecules contained in different solvent boxes. Figure S2. RDF analysis of meropenem charged groups with water: (a) and (b) represent -COO^−^ and -NH_2_^+^ in the water-ethanol system, respectively; (c) and (d) represent -COO^−^ and -NH_2_^+^ in the water-acetone system, respectively. Figure S3. Statistics of the average number of hydrogen bonds formed on one meropenem molecule during molecular dynamics simulation: (a) and (b) between meropenem and water molecules; (c) and (d) between the carboxyl group and water molecules; (e) and (f) between meropenem and the antisolvent. Figure S4. Profiles of turbidity, average chord length, and crystal count distribution within specified chord-length ranges, derived from the frame-by-frame analysis of PAT images in pure water. Figure S5. Profiles of turbidity, average chord length, and crystal count distribution within specified chord-length ranges, derived from the frame-by-frame analysis of PAT images in a solution with an ethanol volume fraction of 20%. Figure S6. Profiles of turbidity, average chord length, and crystal count distribution within specified chord-length ranges, derived from the frame-by-frame analysis of PAT images in a solution with an ethanol volume fraction of 40%. Figure S7. Profiles of turbidity, average chord length, and crystal count distribution within specified chord-length ranges, derived from the frame-by-frame analysis of PAT images in a solution with an ethanol volume fraction of 60%. Figure S8. Profiles of turbidity, average chord length, and crystal count distribution within specified chord-length ranges, derived from the frame-by-frame analysis of PAT images in a solution with a tetrahydrofuran volume fraction of 20%. Figure S9. Profiles of turbidity, average chord length, and crystal count distribution within specified chord-length ranges, derived from the frame-by-frame analysis of PAT images in a solution with a tetrahydrofuran volume fraction of 40%. Figure S10. Profiles of turbidity, average chord length, and crystal count distribution within specified chord-length ranges, derived from the frame-by-frame analysis of PAT images in a solution with a tetrahydrofuran volume fraction of 60%.
