# Supplementary material for: Thermodynamics and Crystallization Behavior of Meropenem Influenced by Solvent Composition and pH
Source: Molecules. 2026 May 28;31(11):1855. doi: 10.3390/molecules31111855 (PMC13257483; doi:10.3390/molecules31111855)
Supplement: Supplementary file 1 [file molecules-31-01855-s001.zip › molecules-4320241-supplementary.pdf]

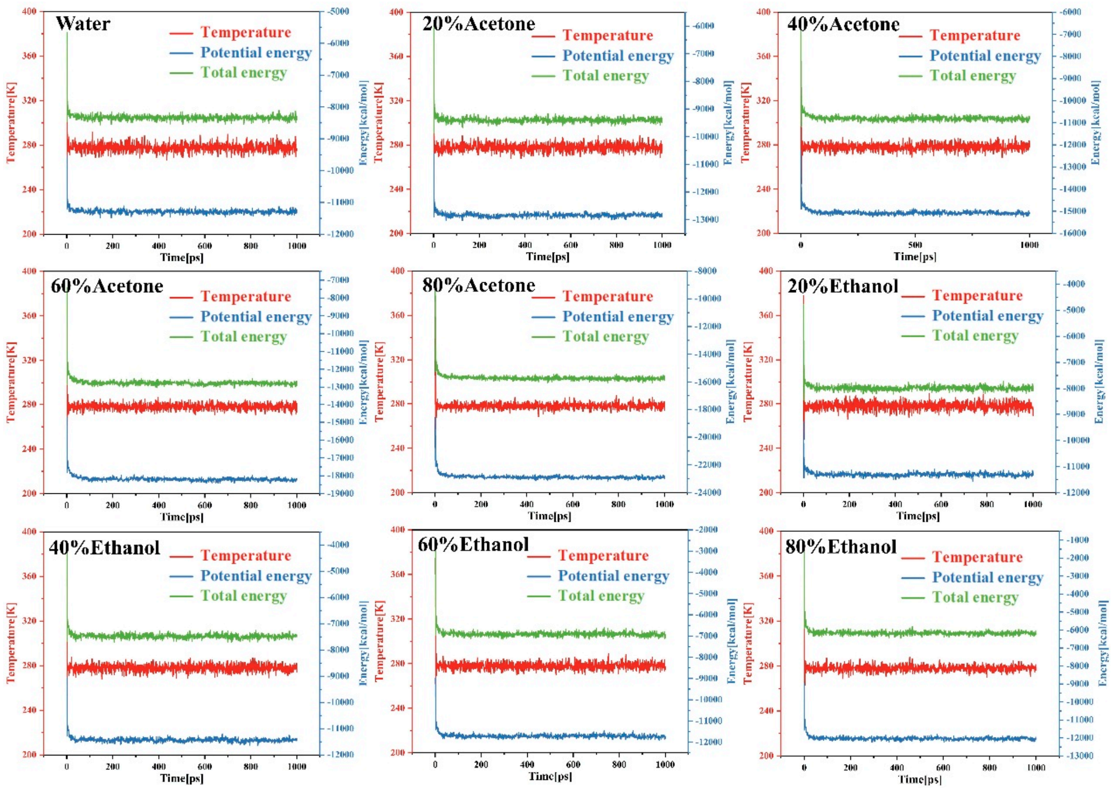

Figure S1. The total energy, temperature and potential energy change over the simulation time.

Table S1. The mole fraction solubility of meropenem trihydrate in water from 278.15 K to 298.15 K determined by experiments and calculated by Tsuji model

pH	10^4x^{exp}	10^4x^{cal}	pH	10^4x^{exp}	10^4x^{cal}
			T=278.15 K		
2.68	8.013	7.753	7.11	4.183	4.475
2.91	6.298	6.157	7.26	4.567	4.723
3.15	4.962	5.187	7.37	4.840	4.968
3.24	4.743	4.941	7.49	5.368	5.317
3.94	4.074	4.086	7.61	5.959	5.777
4.76	3.965	3.907	8.01	8.997	8.658
6.72	4.055	4.118	8.31	14.98	13.42
			T=283.15 K		
2.95	6.912	6.915	5.98	4.579	4.438
3.36	5.344	5.368	6.37	4.627	4.514

3.44	5.219	5.202	6.75	4.790	4.698
3.58	5.086	4.976	7.08	5.016	5.056
3.77	4.794	4.766	7.23	5.254	5.334
4.02	4.675	4.598	7.4	5.723	5.790
4.79	4.471	4.422	7.57	6.882	6.465
		T=288.15 K			
2.85	9.121	9.021	5.8	5.113	5.018
3.14	7.214	7.044	6.35	5.206	5.147
3.38	6.205	6.160	6.67	5.311	5.348
3.60	5.481	5.683	6.84	5.509	5.534
3.83	5.310	5.387	6.99	5.672	5.770
4.26	5.108	5.120	7.74	10.21	9.506
4.84	5.026	5.008	7.91	11.69	11.68
		T=293.15 K			
2.56	17.22	16.26	4.92	5.510	5.459
2.93	10.13	10.03	6.54	5.748	5.786
3.26	7.567	7.568	6.69	5.873	5.944
3.56	6.466	6.488	6.90	6.235	6.281
3.78	5.953	6.057	7.03	6.399	6.587
4.03	5.675	5.771	7.37	7.730	7.994
4.54	5.566	5.519	7.84	13.42	13.05
		T=298.15 K			
2.81	14.91	14.51	5.89	6.665	6.512
2.95	12.27	12.22	6.39	6.837	6.786
3.29	8.953	9.047	6.69	6.903	7.191
3.51	7.715	7.986	7.04	7.519	8.198
3.99	6.752	6.910	7.18	8.353	8.891
4.39	6.576	6.592	7.63	12.55	13.46
4.72	6.502	6.484	7.71	16.24	14.90

Table S2. Experimental and Tsuji model calculated mole fraction solubility of

meropenem trihydrate in water-ethanol at 278.15 K

pH	10^4x^{exp}	10^4x^{cal}	pH	10^4x^{exp}	10^4x^{cal}
		10% Ethanol			
2.48	7.880	7.578	6.73	3.184	3.188
2.64	6.032	6.166	6.93	3.243	3.299
2.81	5.465	5.140	7.35	3.630	3.790
2.95	4.319	4.550	7.57	4.151	4.313
3.30	3.548	3.691	7.82	5.418	5.336
4.06	3.162	3.119	7.99	6.782	6.456
5.01	3.075	3.015	8.30	11.12	10.06
		20% Ethanol			
2.60	4.684	4.531	6.23	2.139	2.273
2.73	3.866	3.936	6.85	2.482	2.409
2.98	3.393	3.189	7.04	2.635	2.508
3.42	2.671	2.577	7.44	2.968	2.931
3.87	2.380	2.353	7.87	4.116	4.117
4.51	2.099	2.258	8.00	4.862	4.776
5.38	1.928	2.239	8.22	6.169	6.457
		30% Ethanol			
2.59	2.941	2.794	6.84	1.569	1.587
2.78	2.228	2.326	7.20	1.724	1.732
3.18	1.726	1.813	7.38	1.841	1.864
3.59	1.644	1.606	7.54	2.072	2.038
4.00	1.448	1.526	7.97	2.928	2.993
5.34	1.312	1.480	8.17	3.945	3.881
5.96	1.395	1.489	8.43	6.862	5.854
		40% Ethanol			
2.57	1.992	1.902	6.47	1.055	1.063
2.69	1.594	1.692	7.15	1.231	1.180
3.04	1.383	1.327	7.58	1.398	1.431
3.57	1.078	1.119	7.79	1.799	1.679
4.07	0.9822	1.060	8.32	3.062	3.223
5.38	0.9213	1.036	8.53	4.957	4.586

5.82	0.9930	1.039	8.79	7.195	7.499
50% Ethanol					
2.64	1.010	0.9581	7.22	0.7424	0.6981
2.71	0.8439	0.9046	7.78	1.061	0.9589
3.18	0.7124	0.7025	8.24	1.530	1.637
4.64	0.6261	0.6028	8.38	2.203	2.032
5.26	0.5918	0.6008	8.60	2.821	2.978
6.55	0.6362	0.6201	8.81	4.632	4.457
60% Ethanol					
2.49	0.9204	0.8991	7.57	0.6985	0.6783
2.67	0.7458	0.7661	7.92	0.8269	0.8902
3.14	0.5549	0.5949	8.19	1.245	1.220
3.81	0.5363	0.5260	8.38	1.556	1.612
6.21	0.5136	0.5147	8.56	2.351	2.179
6.98	0.5541	0.5512	8.81	3.368	3.480
70% Ethanol					
2.37	0.8281	0.7775	7.42	0.5546	0.4967
2.68	0.5432	0.5881	8.03	0.7641	0.7750
3.06	0.5114	0.4820	8.43	1.306	1.332
3.84	0.4946	0.4188	8.86	2.728	2.899
5.48	0.4734	0.4075	9.06	4.590	4.357
6.75	0.5435	0.4256	9.42	9.394	9.458
80% Ethanol					
2.33	0.5431	0.4675	7.75	0.3281	0.3501
2.51	0.3914	0.3925	8.29	0.5542	0.6059
2.94	0.2893	0.3007	8.54	0.8869	0.8856
3.75	0.2480	0.2548	8.79	1.230	1.3831
4.89	0.2316	0.2472	9.09	2.674	2.5144
6.82	0.2963	0.2586	9.40	5.443	4.8770
90% Ethanol					
2.61	0.1084	0.08110	8.94	0.4921	0.4084
3.48	0.06325	0.06868	9.62	1.583	1.731
6.42	0.05842	0.06012	9.76	2.199	2.367

7.58	0.1083	0.07431	9.82	2.955	2.709
8.39	0.1426	0.1575	10.16	6.015	5.857

Table S3. Experimental and Tsuji model calculated mole fraction solubility of meropenem trihydrate in water-acetone at 278.15 K

**pH**	**10^4x^{exp}**	**10^4x^{cal}**	**pH**	**10^4x^{exp}**	**10^4x^{cal}**
		10% Acetone			
2.57	6.882	6.497	6.65	3.323	3.219
2.73	5.229	5.440	7.24	3.618	3.651
3.07	4.191	4.153	7.45	3.889	4.013
3.39	3.478	3.588	7.55	4.363	4.258
3.64	3.355	3.361	7.67	4.781	4.636
4.32	3.212	3.131	8.11	7.299	7.384
5.26	3.150	3.082	8.28	9.079	9.452
		20% Acetone			
2.67	4.580	4.332	6.90	2.732	2.691
2.91	3.481	3.547	7.24	2.877	2.937
3.34	2.913	2.878	7.55	3.413	3.410
3.52	2.687	2.744	7.84	4.461	4.290
3.96	2.581	2.578	8.03	5.057	5.282
4.92	2.426	2.496	8.12	5.912	5.926
5.80	2.575	2.501	8.33	8.320	8.067
		30% Acetone			
2.60	3.311	3.188	6.87	1.992	1.915
2.84	2.450	2.595	7.57	2.417	2.413
3.20	2.122	2.142	8.03	3.623	3.586
3.69	1.914	1.905	8.22	4.348	4.571
4.18	1.870	1.828	8.31	5.396	5.212
4.72	1.786	1.802	8.65	9.827	9.274
5.32	1.755	1.797	8.78	12.20	11.89
		40% Acetone			
2.70	1.637	1.560	7.60	1.538	1.389

2.89	1.334	1.373	7.95	1.941	1.831
3.34	1.156	1.153	8.24	2.792	2.590
4.86	1.053	1.036	8.67	5.194	5.226
6.05	1.106	1.042	8.84	6.930	7.236
6.83	1.227	1.093	8.93	9.391	8.665
7.12	1.316	1.150	9.03	11.74	10.64
			50% Acetone		
2.54	1.568	1.465	7.58	1.138	1.200
2.73	1.182	1.269	7.78	1.224	1.369
3.23	1.049	1.025	8.22	2.493	2.169
3.76	0.9701	0.946	8.57	3.369	3.726
4.49	0.8732	0.9188	8.69	4.911	4.621
6.12	0.7769	0.9225	8.79	5.537	5.581
			60% Acetone		
2.46	1.112	1.014	7.31	0.7159	0.7390
2.72	0.7877	0.8473	7.82	1.040	0.9528
3.46	0.6643	0.6805	8.39	1.648	1.793
4.23	0.5885	0.6498	8.60	2.551	2.508
6.04	0.5167	0.6486	9.09	6.084	6.404
6.95	0.6264	0.6851	9.22	9.042	8.414
			70% Acetone		
2.32	0.6413	0.6417	8.11	0.6839	0.7091
2.68	0.5160	0.4932	8.22	0.8351	0.8045
3.49	0.3885	0.3960	8.77	1.823	1.891
4.22	0.3746	0.3816	8.8	1.932	1.999
5.63	0.2219	0.3794	8.97	2.842	2.775
6.27	0.3543	0.3830	9.17	4.183	4.178
			80% Acetone		
2.53	0.1331	0.1210	8.06	0.2673	0.1443
3.21	0.09263	0.09034	9.02	0.6292	0.6319
4.06	0.08278	0.08511	9.29	1.274	1.104
4.44	0.06929	0.08462	9.48	1.872	1.664
5.21	0.04593	0.08439	9.95	4.682	4.746

7.68	0.1286	0.1071	10.17	7.839	7.820
90% Acetone					
2.68	0.01555	0.01522	7.73	0.01453	0.01646
2.92	0.01443	0.01419	9.75	0.1911	0.3973
3.74	0.01284	0.01300	10.15	0.7719	0.9786
4.86	0.01246	0.01281	10.46	1.740	1.9846
6.42	0.00865	0.01297	10.85	4.924	4.8531

Table S4. The number of meropenem molecules contained in different solvent boxes.

Solvent box	The number of meropenem molecules	Solvent box	The number of meropenem molecules
Water	8	20% Ethanol	5
20% Acetone	5	40% Ethanol	4
40% Acetone	4	60% Ethanol	3
60% Acetone	3	80% Ethanol	2
80% Acetone	2		

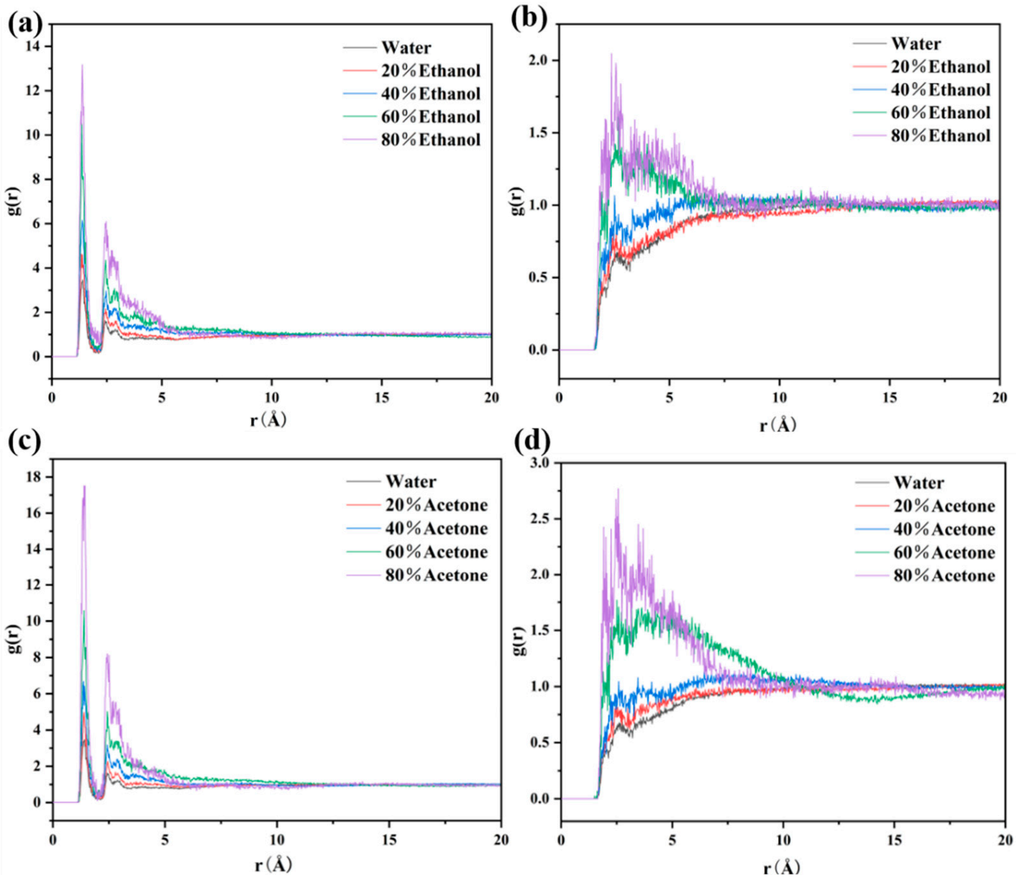

Figure S2. RDF analysis of meropenem charged groups with water: (a) and (b) represent -COO^- and -NH_2^+ in the water-ethanol system, respectively; (c) and (d) represent -COO^- and -NH_2^+ in the water-acetone system, respectively.

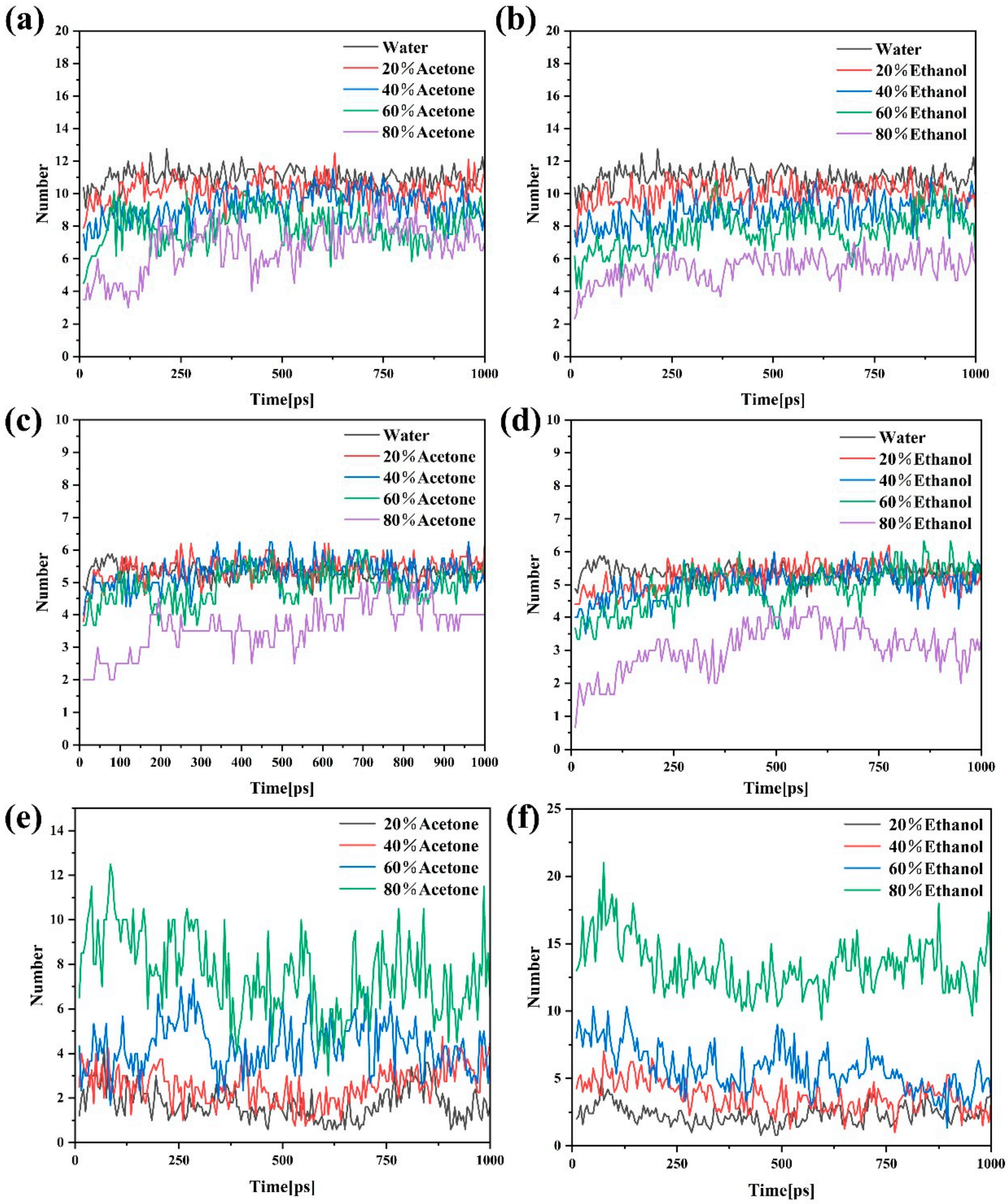

Figure S3. Statistics of the average number of hydrogen bonds formed on one meropenem molecule during molecular dynamics simulation: (a) and (b) between meropenem and water molecules; (c) and (d) between the carboxyl group and water molecules; (e) and (f) between meropenem and the antisolvent.

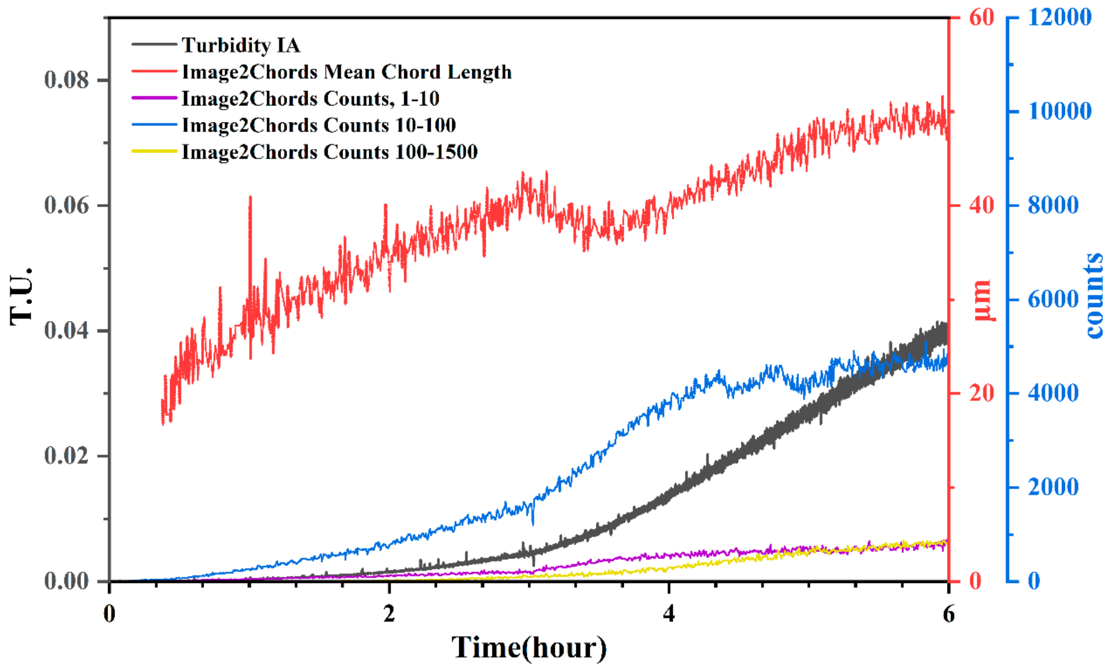

Figure S4. Profiles of turbidity, average chord length, and crystal count distribution within specified chord-length ranges, derived from the frame-by-frame analysis of PAT images in pure water.

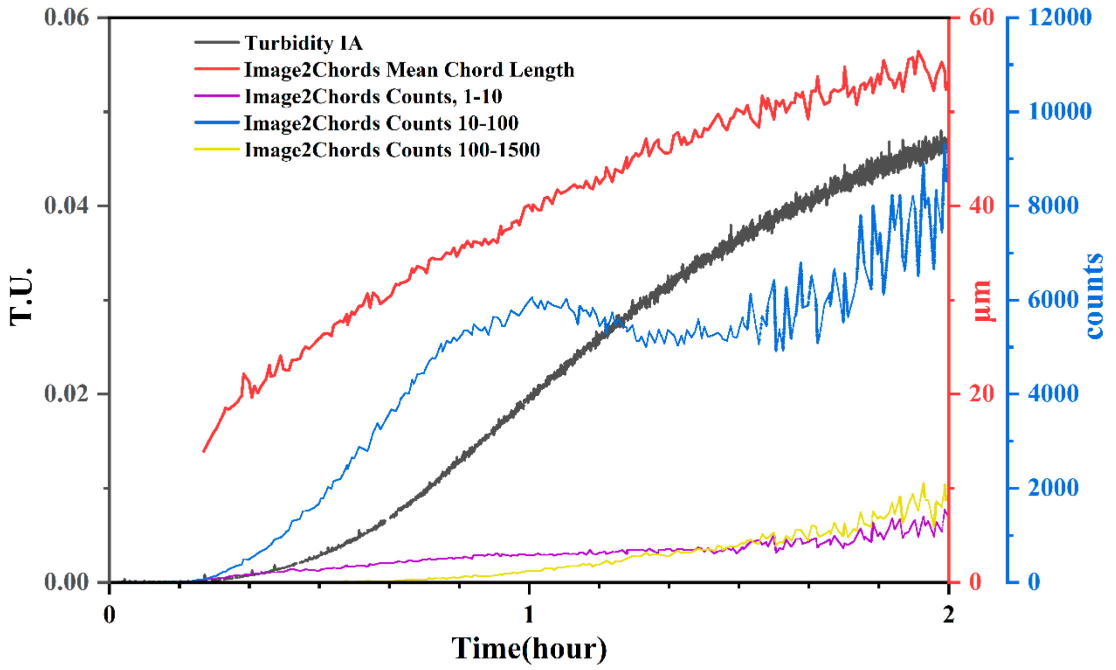

Figure S5. Profiles of turbidity, average chord length, and crystal count distribution within specified chord-length ranges, derived from the frame-by-frame analysis of

PAT images in a solution with an ethanol volume fraction of 20%.

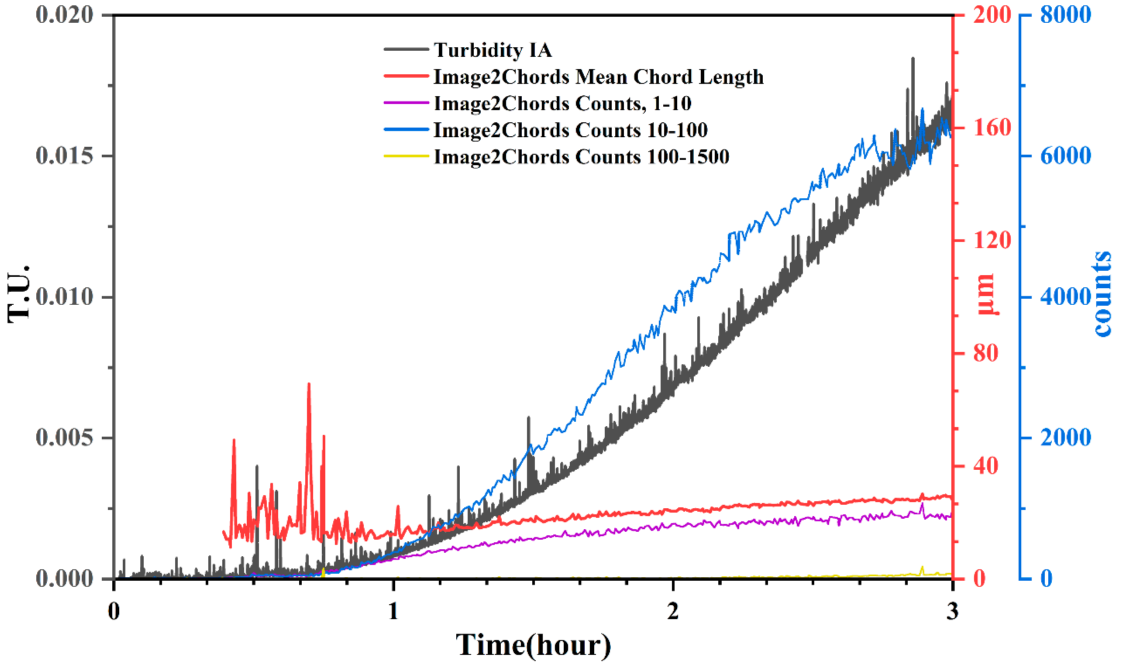

Figure S6. Profiles of turbidity, average chord length, and crystal count distribution within specified chord-length ranges, derived from the frame-by-frame analysis of PAT images in a solution with an ethanol volume fraction of 40%.

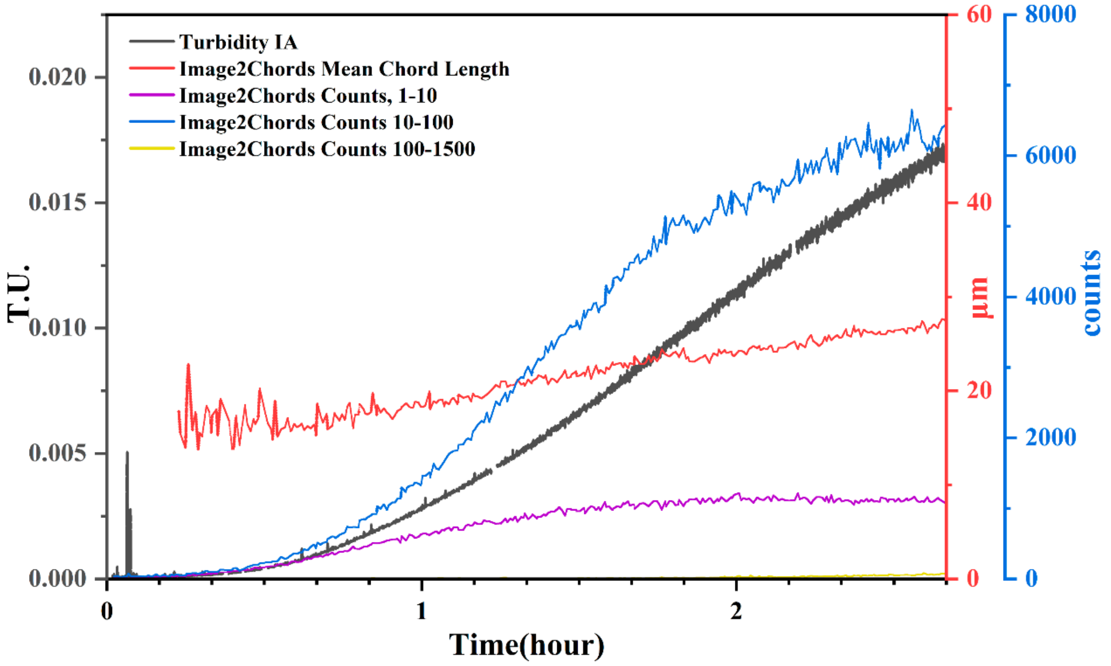

Figure S7. Profiles of turbidity, average chord length, and crystal count distribution within specified chord-length ranges, derived from the frame-by-frame analysis of PAT images in a solution with an ethanol volume fraction of 60%.

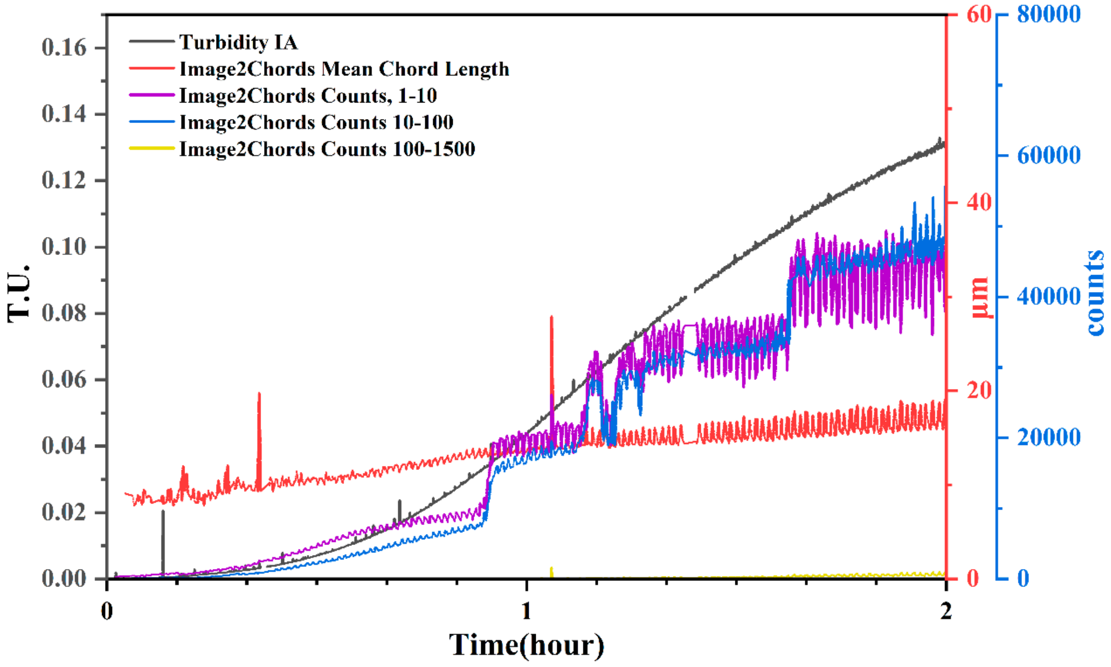

Figure S8. Profiles of turbidity, average chord length, and crystal count distribution

within specified chord-length ranges, derived from the frame-by-frame analysis of PAT images in a solution with a tetrahydrofuran volume fraction of 20%.

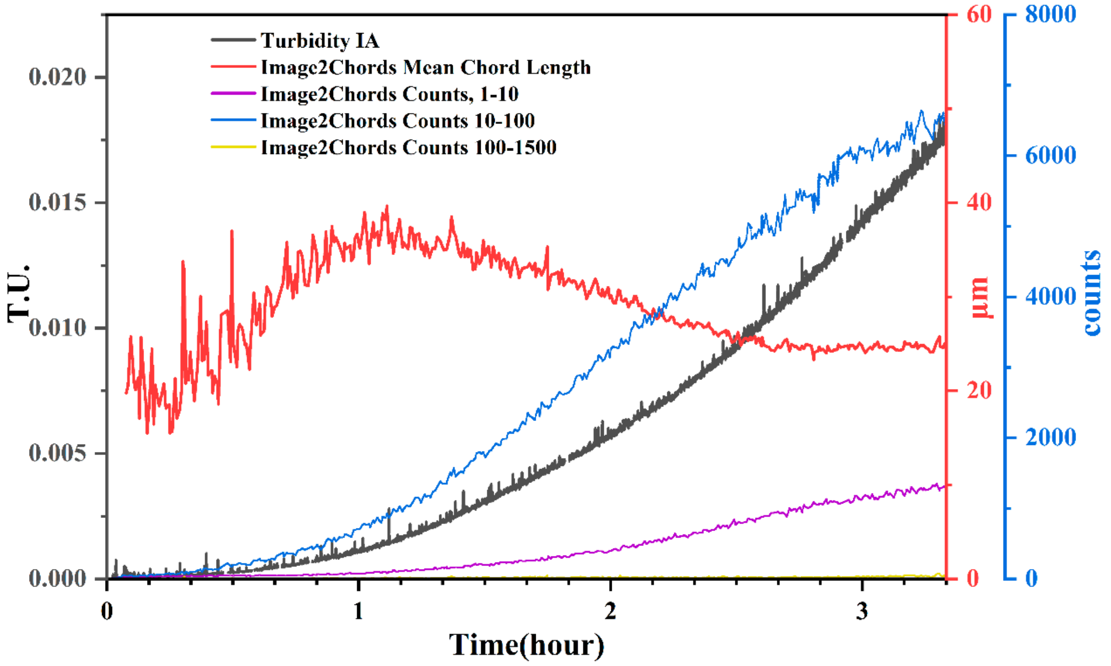

Figure S9. Profiles of turbidity, average chord length, and crystal count distribution within specified chord-length ranges, derived from the frame-by-frame analysis of PAT images in a solution with a tetrahydrofuran volume fraction of 40%.

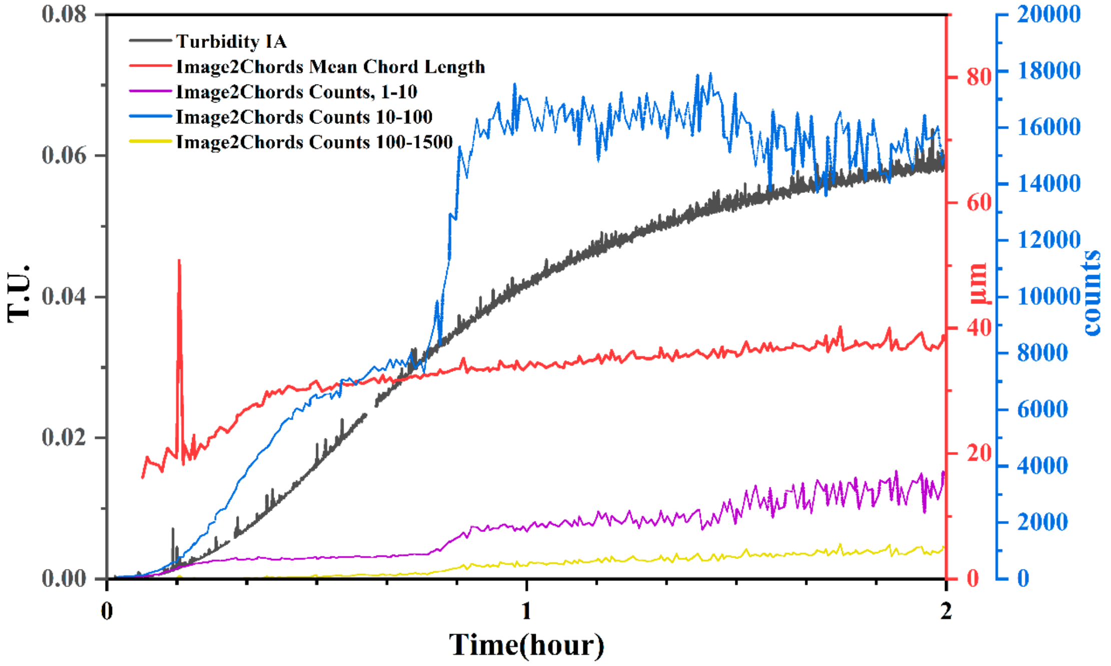

Figure S10. Profiles of turbidity, average chord length, and crystal count distribution within specified chord-length ranges, derived from the frame-by-frame analysis of PAT images in a solution with a tetrahydrofuran volume fraction of 60%.
